# Turban Pin: An Unusual Cause of Foreign Body Aspiration in Young Islamic Adult

**DOI:** 10.5812/ircmj.2975

**Published:** 2014-03-05

**Authors:** Hayriye Gonullu, Yasemin Ozturk, Serhat Akay, Mehmet Boncu, Nazif Erkan

**Affiliations:** 1 Department of Emergency Medicine, Van Yuzuncuyil University, Van, Turkey; 2 Department of Emergency Medicine, Izmir Bozyaka Teaching and Research Hospital, Izmir, Turkey; 3 Department of Chest Disease, Izmir Training and Research Hospital for Thoracic Medicine and Surgery, Izmir, Turkey

**Keywords:** Aspiration, Bronchoscopy, Foreign Bodies

**Figure 1.**
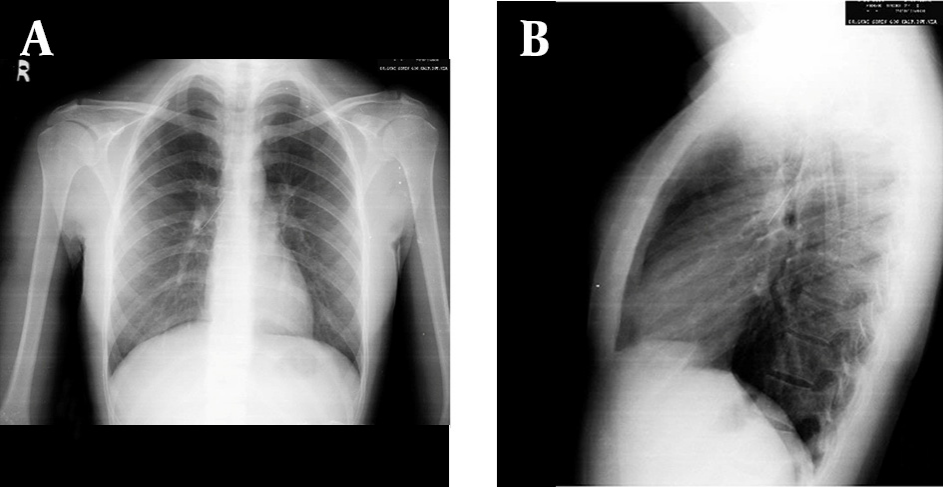
A. PA; B. right lateral Chest X-ray of the Patient Revealing a Radiopaque Foreign Material

Dear Editor,

Foreign body aspiration (FBA) is a common problem among children or infants. Nearly 85% of all FB aspiration occurs in childhood. Common organic aspirated materials include nuts and seeds while inorganic materials include plastic pieces or parts of toys (1). In adults, most of FBAs are seen in the 6th or 7th decade of life when the airway protection mechanism is impaired due to central nervous system dysfunction, intubation or facial trauma (2). Infants are more vulnerable because of immature swallowing coordination and lack of adequate dentition and they like to put objects into their mouths (3). Recently a new cause of FBA associated with traditional or social habits has been described. Turban pin aspirations have been reported in women wearing traditional headscarves in Islamic countries which we try to decribe in this letter. A 17-year-old-woman was referred to our emergency department with turban pin aspiration. Patient had aspirated a turban pin with beads when she tried to talk with the pin in her mouth while fixing the headscarf. She was coughing on admission. Vital signs were normal. No significant abnormality was detected in physical examination. Radioopaque foreign material was visualized with posteroanterior and lateral chest x-rays (Figure 1 A and B). Thoracic surgery department was consulted for urgent bronchoscopy and patient was admitted for further management. The turban pin was extracted with rigid bronchoscopy and forceps under general anesthesia in the operating room. The pin was located in the right main bronchus, about 1 to 2 cm from carina and was impacted in the transverse position. The spherical head of the pin was in the right upper lobe bronchus and the pin was grasped at the middle part and removed by forceps. Repeat chest X-Ray appeared normal and the patient was discharged after 24 hours of observation. There was no complaint or complication during the follow-up.

While wearing turban using their two hands to fix the headscarf, some women prefer to hold the pins between their lips (3). Actions like talking, coughing or laughing may cause aspiration, especially in the inexperienced teenage groups. Unlike other forms of FBA, headscarf pin aspirations are easily diagnosed with chest X-ray because the materials are radio-opaque (4). Patients usually present early as they are frightened and tend to seek medical advice earlier than other forms of aspiration. The chief complaint is usually sudden severe coughing due to irritation of the tracheobronchial tree after aspiration (1, 5, 6). Coughing would be less severe if the pin is localized in the bronchial tree (3). Patients may be asymptomatic initially or present with rales, fever, hemoptysis or dyspnea (1-6). Physical examination findings may vary according to the location of the FBA, time after the aspiration and characteristics of the material (2). Physical examinations of the patient may be normal (as in our case). On the other hand, materials lodged at the laryngeal or subglottic level may cause noisy breathing or hoarseness of voice. Materials lodged in the distal tracheobronchial tree may result in wheezing, cyanosis, subcostal retraction, reduction of breath sounds, rhonchi and high fever. Superimposed infections or sudden death may occur (1). All patients with suspected FBA should receive chest X-ray for visualization and localization of the aspirated material. A negative radiologic finding does not exclude FBA (2, 3, 5). Radio-opaque inorganic materials like turban pin, can easily be localized with posteroanterior and lateral chest X-rays. However, special imaging techniques may be needed to localize radiolucent organic materials (1). Imaging modalities like lateral decubitus chest X-rays, inspiration-expiration chest X-rays and fluoroscopy screenings may be useful to visualize subtle signs not seen in routine imaging. These include mediastinal shift, obstructive emphysema, atelectasis, bronchiectasis and pneumonia (3, 5). Computed tomography may show volume reduction and bronchiectasis in the affected lobe or segment (1). Bronchoscopy is indicated in cases of suspected FBA even if there are no abnormal radiological or clinical signs and symptoms (2). Rigid bronchoscopy is the gold standard for extraction of detected foreign materials (1-3). However, flexible bronchoscopy should be considered as the initial investigation for suspected FBA. Rigid bronchoscopy should be reserved for FBA not extracted by flexible bronchocospy. Direct laryngoscopy and forceps removal may be adequate for FBA lodged in laryngeal or glottic area. Surgical removal is the last resort and is rarely needed. The rate of thoracotomy and bronchotomy in FBA has been reported as 2.5% (1). Turbans are commonly worn in Islamic countries due to religious beliefs. Considering the large number of Islamic women worldwide, turban pin aspiration can become a serious problem. Pin aspiration can be prevented by increasing the public awareness. People should be advised to avoid holding pins in the mouth when fixing a turban. Another solution may be wearing turbans that do not require pins. Different ways to straighten the turban should be found like use of strips or malleable materials. Education of the danger of turban pin aspiration with television or radio broadcast may be useful.
